# Reference values for the appendicular lean muscle index in healthy young Saudi women: a nutritional perspective

**DOI:** 10.3389/fnut.2025.1510432

**Published:** 2025-02-19

**Authors:** Maha H. Alhussain, Samar A. Alamro, Abdullah F. Alghannam, Rawan A. Alabdullatif, Shaea Alkahtani

**Affiliations:** ^1^Department of Food Sciences and Nutrition, College of Food and Agricultural Sciences, King Saud University, Riyadh, Saudi Arabia; ^2^Department of Food Sciences and Human Nutrition, College of Agriculture and Veterinary Medicine, Qassim University, Buraidah, Saudi Arabia; ^3^Lifestyle and Health Research Center, Natural and Health Sciences Research Center, Princess Nourah Bint Abdulrahman University, Riyadh, Saudi Arabia; ^4^Department of Exercise Physiology, College of Sport Sciences and Physical Activity, King Saud University, Riyadh, Saudi Arabia

**Keywords:** sarcopenia, appendicular lean mass, women, bioelectrical impedance analysis, nutrition

## Abstract

**Background:**

Sarcopenia refers to the age-related decline in muscle function, including muscle strength and muscle mass. It can be diagnosed using the appendicular lean muscle index (ALMI) for specific populations. However, reference values for the ALMI in Saudi women are lacking.

**Aim:**

This study aimed to determine the ALMI reference values in young Saudi women using bioelectrical impedance analysis (BIA). The relationship between ALM and dietary intake was also investigated.

**Methods:**

A total of 387 healthy young Saudi women, aged between 18 and 25 years, were included in this study. They were recruited through a convenience sampling method between October 2020 and June 2021. Body composition was assessed using BIA (Inbody 770). The ALMI was determined by calculating the sum of lean tissue in the arms and legs and dividing it by the height squared (ALM/h^2^). The cutoff value was calculated by deriving the −2 standard deviation (SD) value based on the participants’ data. A 24-h dietary recall was also conducted, and energy and macronutrient intakes were assessed.

**Results:**

The mean ALM/h^2^ was 5.63 ± 0.77 kg/m^2^, and the mean ALM/ht^2^, −2 SD value was 4.09 kg/m^2^ for the young Saudi reference group. There were significant positive correlations between ALM and protein (g/day) (*r* = 0.15; *p* < 0.001), protein (%) (*r* = 0.16; *p* = 0.002), fiber (g/day) (*r* = 0.21; *p* < 0.001), and cholesterol (mg/day) (*r* = 0.14; *p* = 0.007). However, the ALM showed a significant negative correlation with carbohydrates (%) (*r* = − 0.11; *p* = 0.03).

**Conclusion:**

These findings provide valuable reference values for evaluating ALM in patients with a variety of diseases that impact ALM. Furthermore, a cutoff value for low ALM may assist in the diagnosis of sarcopenia in Saudi women and enhance our understanding of the effects of total dietary nutrient intake on sarcopenia.

## 1 Introduction

The global population of adults aged 60 years and older is growing more rapidly than that of younger age groups. It is predicted that this population will more than double by 2050 and more than triple by 2,100, increasing from 962 million in 2017 to 2.1 billion in 2050 and 3.1 billion in 2100 (1). Population aging is expected to have numerous consequences, including increased household costs, strains on public finances and healthcare services, and decreased economic growth. Sarcopenia, an age-related decline in muscle function, including muscle strength and muscle mass (2), is one of the major health concerns associated with aging. It can lead to significant clinical implications such as increased frailty, poor quality of life, and higher rates of hospitalization (3, 4). A multi-dimensional approach to diagnosing sarcopenia involving the assessment of muscle mass, muscle strength, and physical performance has been proposed (5, 6). The appendicular lean mass index (ALMI) is a useful metric employed to assess muscle mass. The ALMI is calculated by dividing the appendicular lean mass (the total lean mass of the four limbs) by height squared (ALM/height^2^). The European Working Group on Sarcopenia in Older People 2 (EWGSOP2) defined the following reference values for the ALMI based on gender-specific cutoff values: men: ≤7.23 kg/m^2^ and women: ≤5.67 kg/m^2^. These cutoff values are indicative of low muscle mass and are used for diagnosing sarcopenia (7). These values are derived from studies primarily conducted on European populations. Research conducted on Asian populations has recommended different cutoff values for the ALMI compared to those proposed by the EWGSOP2 (8). Indeed, body composition can vary significantly across different ethnic groups, which can, in turn, influence the diagnosis and management of sarcopenia. Therefore, it is recommended to use reference values based on healthy young adults from a specific population, rather than relying on values predicted from a reference population. A study (9) involving 232 young Saudi men aged 20 to 35 years reported the following reference values for the ALMI in the Saudi population: 8.97 ± 1.23 and 8.16 ± 0.87 kg/m^2^, measured using whole-body dual-energy X-ray absorptiometry (DXA) and bioelectrical impedance analysis (BIA), respectively. Aljawini and Habib also conducted a study on 59 Saudi women (mean age: 42.63 ± 8.25 years) and reported a value of 7.47 ± 1.07 kg/m^2^ using BIA (10). However, the values reported in their study were derived from a small number of participants. To expand on these findings and establish more precise ALMI cutoff values for diagnosing sarcopenia in Saudi women, further research with a larger cohort of young, non-sarcopenic Saudi women is needed.

While the exact etiology of sarcopenia remains unclear, it is believed to be multifactorial, with a combination of biological, environmental, and lifestyle factors (e.g., poor diet) contributing to its development (11). A lack of adequate energy and essential nutrient intake can lead to changes in body composition that are characterized by muscle mass loss (12). Nutrient intake, particularly protein, markedly impacts muscle mass. The balance between muscle protein synthesis and muscle protein breakdown is a major determinant of muscle mass (13). Reduced energy intake causes a reduction in protein synthesis (14). Our previous study showed that adult men with normal muscle mass have greater daily energy, protein, and fat intakes and a lower percentage of energy from CHO, compared to sarcopenic individuals (15).

The primary aim of the current study was to determine ALMI reference values in young Saudi women using a BIA device. The relationship between the ALMI and dietary intake was also investigated in this study.

## 2 Methods

### 2.1 Study design and participants

A total of 387 young Saudi women participated in this descriptive, cross-sectional study. A convenience sample of students was recruited through advertisements at King Saud University between October 2020 and June 2021. The eligibility criteria for this study included being of Saudi nationality, aged between 18 and 25 years, in good health, with no physical or mental diseases, and having normal muscle mass, as confirmed through BIA. The exclusion criteria were pregnant/lactating women, professional athletes, individuals with chronic diseases such as cardiovascular disease, diabetes, hypertension, renal or hepatic disease, and those taking medications that may affect body composition.

This study was conducted in accordance with the Declaration of Helsinki established by the World Medical Association and received approval from the Internal Review Board at King Saud University, Riyadh, Saudi Arabia. The participants voluntarily signed consent forms before their participation in the study.

### 2.2 Measurements

Potential participants were asked to arrive at the laboratory by 08:00 a.m. after overnight fasting for ≥8 h. Hydration status and exercise levels were monitored to ensure reliable BIA measurements. Upon arrival at the laboratory, the investigator verified the eligibility of the participants. Anthropometric measurements, body composition and dietary intake data were then collected from the participants who met the eligibility criteria.

#### 2.2.1 Anthropometrics

All measurements were recorded following the standard protocol by a trained investigator. Height was measured to the nearest 0.5 cm using a standing scale while the participant was standing fully upright and without shoes. Weight was measured using a BIA device (InBody 770, Inbody Co., Ltd., Seoul, Republic of Korea) with the participants having an empty bladder, wearing light clothing, and no shoes. The participants stood on a balance scale barefoot and held a pair of conductive handles, one in each hand. After the 15-s measurement, the output, including body weight, was printed. Body mass index (BMI) was calculated as weight divided by the square of height. Waist and hip circumferences were measured to the nearest 0.5 cm using a non-stretchable measuring tape, and then, the waist-to-hip ratio (WHR) was calculated.

Body composition was assessed using BIA according to the manufacturer’s guidelines. Visceral fat, body fat percentage (BF%), and skeletal muscle mass (SSM) were obtained through body composition analysis. The ALMI was calculated by dividing ALM (the total amount of lean tissue in the arms and legs) by height squared (ALM/h^2^) (16). Additionally, ALM divided by the BMI was also calculated. The cutoff value was defined as two standard deviations below the mean value for the young reference group, as recommended by the EWGSOP2 (6) and the Asian Working Group for Sarcopenia (AWGS) (8).

### 2.3 Dietary intake

The participants were interviewed face-to-face by a trained researcher to collect a 24-h dietary recall for a typical day. Prior to the recall, the participants were informed about the process and were encouraged to remember all foods and drinks they consumed. To improve the accuracy of the dietary intake data, the USDA five-step multiple-pass method was employed (17). This method involved a quick free recall of all foods and beverages consumed, followed by targeted probing to account for forgotten items, along with details on portion sizes and preparation methods. The participants were provided with visual aids, such as food models and portion size guides, to assist them in estimating the quantities consumed. Dietary data were analyzed using Food Processor software (ESHA Research, Inc., Salem, OR) (18) to calculate the daily energy and macronutrient intake.

### 2.4 Statistical analysis

SPSS software (Version 28; SPSS Inc.; Chicago, IL, USA) was used for data entry and analysis. The data were presented as means with their standard deviations (SD). The Kolmogorov–Smirnov test was used to verify the data for normality. Pearson’s correlation coefficient was computed to assess the relationship between ALM and anthropometrics, body composition, and dietary intake. Correlations were classified as weak if *r* < 0. 5, moderate if *r* ≥ 0.5 to <0.8, strong if *r* ≥ 0.8, and perfect if r = 1 (19). An independent samples *t*-test was performed to compare the ALMI between overweight/obese participants and non-overweight/obese participants. Statistical significance was set at a *p*-value of <0.05 for all statistical tests.

## 3 Results

**Figure 1:**
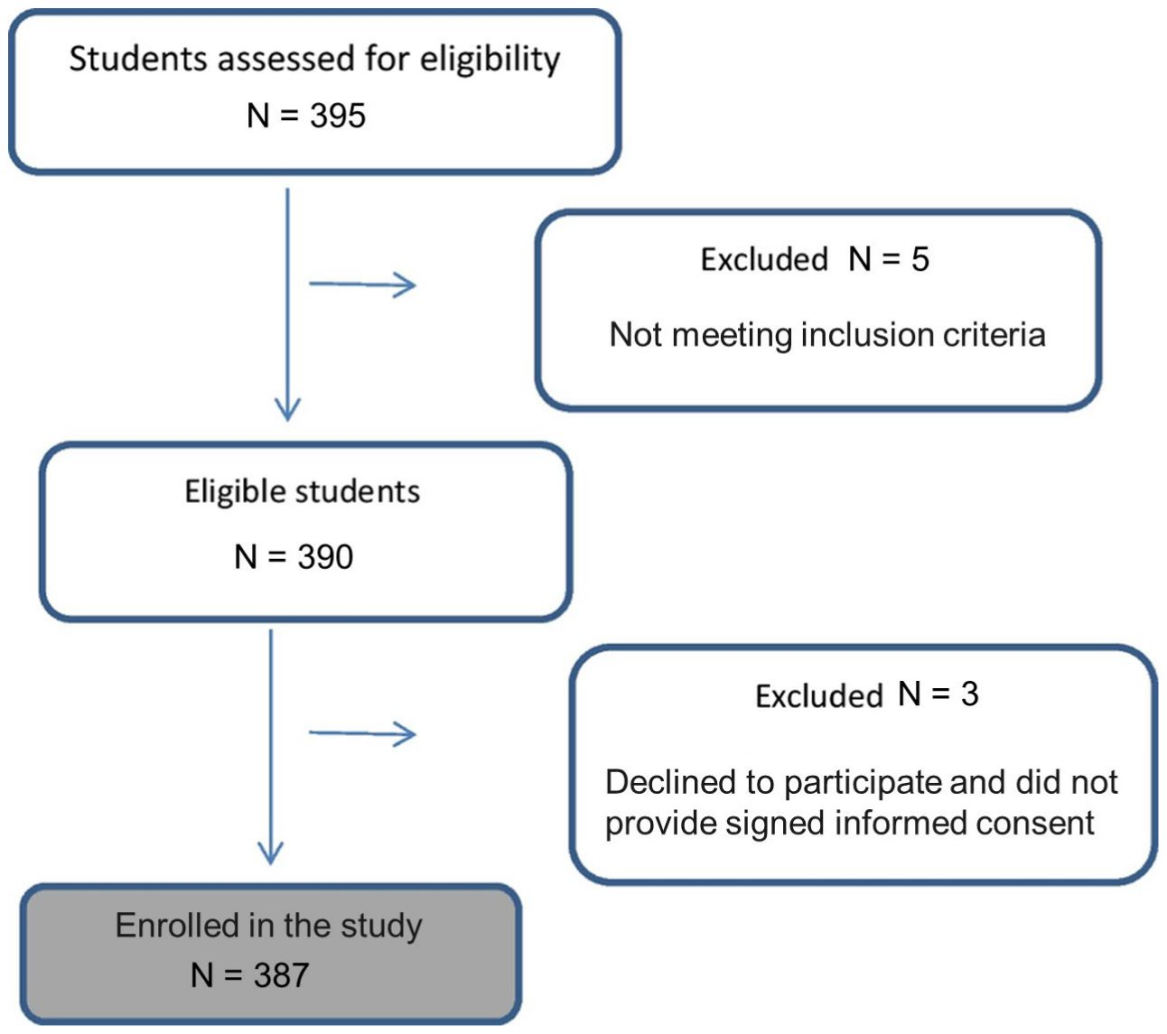
Flowchart of study enrollment.

Of the 395 participants assessed for eligibility, 8 were excluded and 387 were enrolled and included in the final analysis (Figure 1). They were considered the sex-specific young reference group of women in Saudi Arabia. The descriptive characteristics of the participants are detailed in Table 1. The participants were, on average, 20.7 ± 1.6 years old, with a normal BMI of 22.9 ± 4.9 kg/m^2^. The mean ALM and ALM/ht^2^, as well as one and two SDs below the mean value for the young reference group, are illustrated in Table 1.

**Table 1:** Descriptive characteristics of the study participants (*n* = 387).

Characteristics	Mean ± SD
Age (years)	20.7 ± 1.6
Anthropometrics	
Height (cm)	158.2 ± 5.6
Weight (kg)	57.6 ± 12.8
BMI (kg/m^2^)	22.9 ± 4.9
WC (cm)	63.4 ± 9.1
HC (cm)	97.7 ± 10.8
WHR	0.7 ± 0.1
Body composition	
Visceral fat (cm^2^)	108.9 ± 50.1
Body fat (%)	36.6 ± 7.7
SMM (kg)	19.1 ± 2.9
ALM (kg)	14.2 ± 2.4
ALM/BMI	0.6 ± 0.1
ALM/h^2^ (kg/m^2^)	5.63 ± 0.77
-1 SD	4.86
-2 SD	4.09

BMI, body mass index; WC, waist circumference; HC, hip circumference. WHR Waist-to-hip ratio; SMM, skeletal muscle mass; ALM, appendicular lean mass.

**Figure 2:**
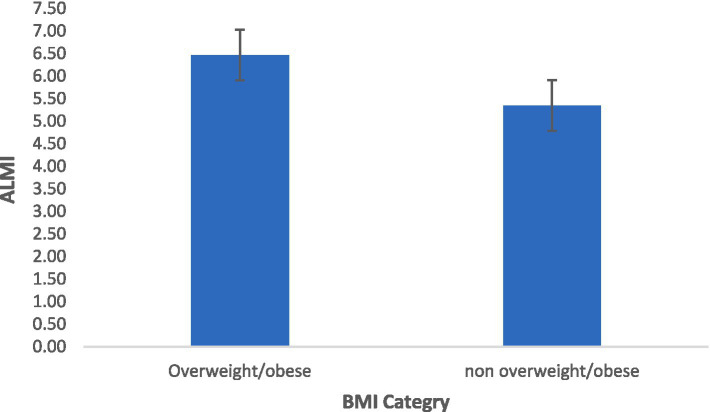
Mean values of the ALMI based on the BMI category: overweight/obese participants (*n* = 100) and non-overweight/obese participants (*n* = 287). A significant difference between the two BMI categories was observed, *p*-value <0.001 (independent samples *t*-test).

Figure 2 shows the mean values of the ALMI based on the BMI category. Overweight/obese participants had a significantly higher ALMI value compared to non-overweight/obese participants (6.5 ± 0.7 vs. 5.3 ± 0.6; *p* < 0.001, the independent samples *t*-test).

The intake of calories and macronutrients is shown in Table 2. The average energy intake of all participants was 1335.1 ± 409.9 Kcal/day.

**Table 2:** Intake of calories and macronutrients of the study participants (*n* = 387).

Characteristics	Mean ± SD
Total energy intake (Kcal/day)	1335.1 ± 409.9
Carbohydrate (g/day)	157.8 ± 56.9
Carbohydrate (energy%)	47.5 ± 10.5
Fiber (g/day)	7.6 ± 5.7
Protein (g/day)	52.3 ± 24.3
Protein (energy%)	15.7 ± 6.2
Fat (g/day)	57.3 ± 24.2
Fat (energy%)	38.3 ± 9.9
Total (mg/day)	188.8 ± 146.1

Table 3 displays the Pearson correlation between ALM and the study variables. ALM showed significant positive strong correlations with weight and BMI (*p* < 0.001). Furthermore, ALM showed significant positive moderate correlations with WC, HC, and visceral fat (*p* < 0.001). In terms of dietary intake, there were significant positive correlations between ALM and protein (g/day) (*p* < 0.001), protein (%) (*p* = 0.002), fiber (g/day) (*p* < 0.001), and cholesterol (mg/day) (*p* = 0.007). On the other hand, ALM showed a significant negative correlation with carbohydrates (%) (*p* = 0.03).

**Table 3:** Pearson correlation coefficient of ALM with the study parameters.

Parameters	r	(95% CI)	*P*-value
Age	0.06	−0.04 - 0.16	0.22
Weight	0.87	0.84–0.89	**< 0.001**
Height	0.29	0.2–0.38	**< 0.001**
WC	0.74	0.69–0.78	**< 0.001**
HC	0.76	0.71–0.8	**< 0.001**
BMI	0.82	0.78–0.84	**< 0.001**
Visceral fat	0.61	0.55–0.67	**< 0.001**
BF	0.48	0.41–0.54	**< 0.001**
Total energy intake (Kcal/day)	0.10	0–0.19	0.06
Protein (g/day)	0.15	0.05–0.25	**< 0.001**
Protein (%)	0.16	0.06–0.26	**0.002**
Carbohydrates (g/day)	0.02	−0.08 - 0.12	0.75
Carbohydrates (%)	- 0.11	−0.2 - -0.02	**0.03**
Fiber (g/day)	0.21	0.11–0.3	**<0.001**
Fat (g/day)	0.09	0.01–0.17	0.051
Fat (%)	0.05	−0.04 - 0.14	0.29
Cholesterol (mg/day)	0.14	0.05–0.23	**0.007**

The data represent the coefficient of determination. The p-value is significant at both the 0.01 and 0.05 levels. Bold values indicate significance.

## 4 Discussion

The prevalence of sarcopenia varies based on different cutoff values, which are influenced by several factors, including ethnicity and reference populations. It has been recommended that the ALMI should ideally be based on sex-specific ethnic groups for greater accuracy and relevance. The present study aimed to establish a reference value that can be locally used to diagnose sarcopenia based on muscle mass. Determining the mean values of the ALMI for young Saudi reference groups is crucial for guiding future research in Saudi Arabia. This will help identify cutoff points related to muscle mass and understand the modifiable lifestyle factors affecting young people, ultimately aiding in the prevention or delay of sarcopenia.

In the current study, the mean sex-specific ALM/h^2^ value for healthy young Saudi women, measured using BIA, was 5.63 ± 0.77 kg/m^2^. This value was similar to that found in other Asian populations. For instance, in Korean counterparts in their twenties, the value was reported to be 5.64 kg/m^2^ (20). In contrast, a previous local study conducted on Saudi women (mean age: 42.6 ± 8.3 years; mean BMI: 31.5 ± 5.6 kg/m^2^) reported a value of 7.47 ± 1.07 kg/m^2^ (specifically, the value for obese women was 8.17 ± 0.88, while for non-obese women with a BMI of 26 kg/m^2^, it was 6.80 ± 0.76 kg/h^2^) (10). The high value reported in the previous study in Saudi women, compared to Asian studies, highlights the importance of our current study in determining whether this variation is due to ethnic differences or measurement limitations. The primary reason for the difference between our results and those reported in the previous local study (10) may be attributed to variations in anthropometric factors, particularly BMI. Although the average age in the previous study was over 42 years, we do not consider age to be the reason for the higher value in their study. It should be noted that muscle mass increases during young adulthood, reaching its peak around the age of 30 years. After this peak, muscle mass gradually declines (6, 21).

In the current study, the cutoff reference value for Saudi women was 4.4 kg/m^2^. This cutoff was determined using the suggested values of two standard deviations below the sex-specific means for young adults, as adopted by the EWGSOP2 (6) and the AWGS (8). In line with our findings, a study conducted on Indian women reported that the cutoff value (−2 standard deviation) for ALM was 4.42 kg/m^2^ (22). Another study from China revealed that the cutoff value (−2 standard deviation) for ALM in Chinese women was 4.79 kg/m^2^ (23). Different cutoff values for sarcopenia have been reported by the AWGS, the International Working Group on Sarcopenia (IWGS), and the EWGSOP2. For women, the ALM/h^2^ cutoff values, measured using BIA, are as follows: EWGSOP2 < 6.0 (6), IWGS ≤5.67 (24), and AWGS <5.4 kg/m^2^ (8). Therefore, for Saudi women, the ALM/h^2^ cutoff values proposed by the IWGS or AWGS are acceptable.

Measuring muscle mass can help identify individuals at risk for muscle decline and adverse health outcomes, and interventions can be targeted to mitigate these risks and improve outcomes in older adults. A wide range of techniques are available to assess muscle mass. Although whole-body dual-energy X-ray absorptiometry (DXA) is precise and recommended (25), muscle mass can be validated through other techniques to enhance its operationalization and applicability in clinical and research settings. The EWGSOP and AWGS considered BIA to be an effective portable alternative to DXA (5, 8). BIA is a non-invasive, quick, and inexpensive technique for measuring body composition, and its results have been found to correlate well with DXA (25) and MRI predictions (26).

We studied the relationships between ALM and dietary nutrient intake and found that ALM was positively correlated with protein intake. Protein has been recommended as a key component in the treatment of sarcopenia (27, 28). Inadequate protein intake may contribute to a loss of muscle mass and strength due to a prolonged imbalance between muscle protein synthesis and breakdown (29). Previous research has reported similar findings, with reduced protein consumption observed in sarcopenic versus non-sarcopenic older adults (30, 31). In this study, we observed that ALM had a significant positive correlation with fiber and cholesterol. On the other hand, ALM showed a significant negative correlation with the percentage of carbohydrates. Increased carbohydrate intake may be a result of insufficient protein intake. Thus, monitoring food composition may be important (15).

Preserving lean muscle mass and strength throughout life is critical to maintaining health as skeletal muscles are important for cardiometabolic health and functional capacity (32). However, sarcopenia remains overlooked and undertreated (33, 34), presumably due to the challenges in determining which variables to measure, how to measure them, and what cutoff points to use to best guide diagnosis and treatment (35). Cutoff points for detecting and diagnosing sarcopenia depend on the techniques used and the availability of reference studies and populations. It is recommended that normative references for a population be based on the values of healthy young adults (36) to accurately diagnose sarcopenia. An important factor in determining the magnitude of muscle mass and strength and the decline in these parameters is ethnicity (37, 38). Therefore, due to hereditary factors, ethnicity, and body size, international criteria for sarcopenia detection and diagnosis may not apply to Saudi individuals, highlighting the importance of region-specific reference values. This is exceedingly important as Saudi women have been shown to have a low level of physical activity (39, 40).

This study has some strengths, as it may be the first ethnic study to establish a reference value for young Saudi women, which is greatly needed in the clinical context. Moreover, including a nutritional perspective will help guide researchers and clinicians in prioritizing nutritional and lifestyle approaches. On the other hand, the study was limited to evaluating muscle mass; muscular function, such as strength and performance, was not assessed. Furthermore, the sample size was from a single site and did not include the rural population of Saudi Arabia. Multicenter research is required to adequately represent the geographical and socioeconomic diversity of Saudi Arabia.

## 5 Conclusion

The mean value for ALM/h^2^ in young healthy Saudi women, measured using BIA, was 5.63 ± 0.77 kg/m^2^, which differs from previously reported values for other ethnicities and geographic locations. These findings provide useful reference values for assessing ALM/h^2^ in patients with a variety of diseases that affect ALM/h^2^. Furthermore, a cutoff value for low ALM may help in the diagnosis of sarcopenia in Saudi women and enhance our understanding of the effects of total dietary nutrient intake on sarcopenia in adult women. Future studies should prioritize the validation of the proposed cutoff values by exploring their association with clinical outcomes, including various diseases, inflammatory markers, and mortality risk. This would help establish these thresholds as clinically relevant in practical settings. Moreover, future research should include assessments of muscle strength and performance to provide a more comprehensive understanding of muscle health.

## Data Availability

The raw data supporting the conclusions of this article will be made available by the authors, without undue reservation.
